# Self-directed learning can outperform direct instruction in the course of a modern German medical curriculum - results of a mixed methods trial

**DOI:** 10.1186/s12909-016-0679-0

**Published:** 2016-06-03

**Authors:** Arne Peine, Klaus Kabino, Cord Spreckelsen

**Affiliations:** Department of Medical Informatics, RWTH Aachen University, Faculty of Medicine, Pauwelsstr. 30, 52074 Aachen, Germany

**Keywords:** Curriculum development, Germany, Self-instructed learning, E-Learning, Medical student, Medical school, Learning style

## Abstract

**Background:**

Modernised medical curricula in Germany (so called “reformed study programs”) rely increasingly on alternative self-instructed learning forms such as e-learning and curriculum-guided self-study. However, there is a lack of evidence that these methods can outperform conventional teaching methods such as lectures and seminars.

This study was conducted in order to compare extant traditional teaching methods with new instruction forms in terms of learning effect and student satisfaction.

**Methods:**

In a randomised trial, 244 students of medicine in their third academic year were assigned to one of four study branches representing self-instructed learning forms (e-learning and curriculum-based self-study) and instructed learning forms (lectures and seminars). All groups participated in their respective learning module with standardised materials and instructions. Learning effect was measured with pre-test and post-test multiple-choice questionnaires. Student satisfaction and learning style were examined via self-assessment.

**Results:**

Of 244 initial participants, 223 completed the respective module and were included in the study. In the pre-test, the groups showed relatively homogenous scores. All students showed notable improvements compared with the pre-test results. Participants in the non-self-instructed learning groups reached scores of 14.71 (seminar) and 14.37 (lecture), while the groups of self-instructed learners reached higher scores with 17.23 (e-learning) and 15.81 (self-study). All groups improved significantly (*p* < .001) in the post-test regarding their self-assessment, led by the e-learning group, whose self-assessment improved by 2.36.

**Conclusions:**

The study shows that students in modern study curricula learn better through modern self-instructed methods than through conventional methods. These methods should be used more, as they also show good levels of student acceptance and higher scores in personal self-assessment of knowledge.

**Electronic supplementary material:**

The online version of this article (doi:10.1186/s12909-016-0679-0) contains supplementary material, which is available to authorized users.

## Background

Over the past 10 years, teaching approaches for medical students at German universities have greatly changed. Instead of conventional programs relying on traditional teaching methods, many universities have developed so-called ‘reformed study programs’ (“Modellstudiengänge”). In 2015, around one-third of medical faculties in Germany offered these programs to their students: some offered them exclusively, and some offered them in addition to the normal degree programmes.

An essential characteristic of these programmes is the increased use of alternative and new instructional methods, such as PBL (problem-based learning) [1] in small groups, e-learning courses or case-based work on issues in small working groups. Yet classic elements are still present in most faculties, such as lectures and self-instructed learning. Study progress is also assessed by means of newly introduced testing methods like Objective Structured Clinical/Practical Examination (OSC/PE) and Modified Essay Questions Tests (MEQ).

Alternative concepts of traditional teaching, like blended learning and web-based training, are broadly used in the field of medical education. Finding the ideal form to provide students with sustainable knowledge is the subject of several different studies. Although there are many studies comparing two or more instruction forms [2–4], there are no studies that compare traditional teaching methods to self-directed learning within the course of a modern medical curriculum.

Previous studies compared various forms of academic learning, including web-based learning, problem-based learning or instruction in small groups. These studies usually focus on two individual elements in terms of efficiency: student satisfaction and learning outcomes [5–9]. A study comparing learning outcomes and student satisfaction in a classroom setting versus an online instructional course does not show significant differences in learning outcomes, but reveals generally high acceptance and student satisfaction levels [5].

The same finding is also supported by a 2002 literature-based review on 35 research papers comparing traditional teaching methods with web-based learning (WBL). The review concluded that acceptance is generally high, but there is no evidence for the superiority or inferiority of WBL [10].

Other universities are using e-learning based clinical pharmacology courses with great success. A 2013 report from TU München showed “efficient generation of high-quality e-learning content in a student-centred and interdisciplinary manner” [11] and high acceptance by the students. A 2004 study compared self-directed teaching with traditional lecture-based teaching in out-of-hospital training. This study showed “modest improvement in cognitive evaluation scores over traditional teaching when measured at the end of the course” [8].

In Germany, students are free to choose whether they want to study a traditional degree programme or a reformed study programme. There is a lack of systematic studies on whether students in reformed study programmes can attain higher achievements by using specific forms of teaching. It could be hypothesised that students in modern study programmes prefer more independent and self-instructed learning methods.

Our study compares the effect of four teaching interventions – including non-guided, self-directed learning – in the context of a medical model curriculum.

Particular emphasis was placed on learning outcomes, exam scores, self-assessments, student satisfaction and students’ long-term learning effects. Although in our faculty each course is evaluated by itself after completion, thus far no systematic comparisons between these forms of learning have been made.

### The Aachen reformed study program (“Aachener Modellstudiengang”)

The Rheinisch-Westfälische Technische Hochschule (RWTH) University in Aachen, Germany has approximately 40,000 enrolled students, including approximately 2000 students in the medical faculty in 2015. The Faculty of Medicine established the modernised, ‘reformed’ model curriculum in 2003. It was entirely redesigned and the six-year programme now consists of three major sections.

In the first section (first year), theoretical sciences are taught primarily. In the second section, a clinical-theoretical training is taught in so-called ‘system blocks’. These learning modules are 6–8 weeks long, and each deals with one topic in an interdisciplinary context. These multidisciplinary system blocks are the backbone of the system-centred Aachen curriculum. During an interval of 4–6 weeks, they focus on a special organ system and combine the contributions of different medical specialties teaching different aspects of a topic, e.g. anatomy, physiology, pathology and pharmacology. System blocks enclose various forms of teaching (lectures, PBL, seminars, e-learning, practical demonstrations, etc.). A final examination closes each block, which is then followed by the next block. Passing the final exam is mandatory for admission to the third study section. This section discusses relevant diseases on the basis of symptoms (i.e. ‘headache’, ‘vertigo’) and leads the students from the diagnosis of the disease to its treatment [12].

Since its establishment in 2003, all students at the Medical Faculty - excepting the students of dentistry - are enrolled in this curriculum. Approximately 250 students successfully complete this programme each year, including the state examination.

## Methods

### Study design and standardisation

**Fig. 1:**
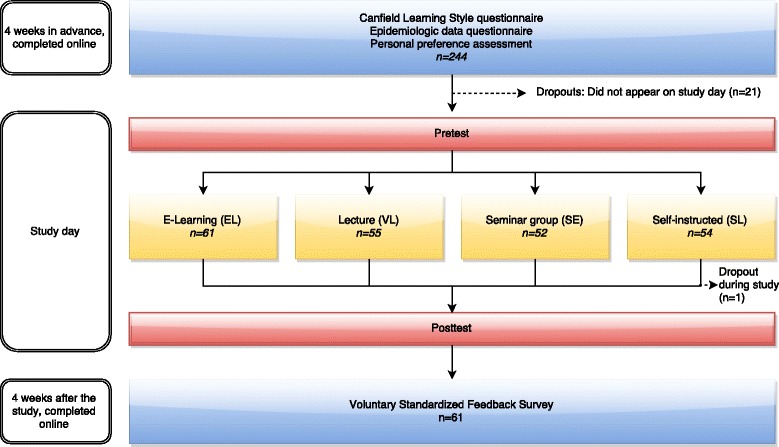
Study flowchart

Our study compares four established learning forms at our faculty within a well-tested, sealed environment: a one-day pharmacology course with all third-year students involved. Some questionnaires were completed online before and after the study. A detailed flowchart of the study is shown in Fig. 1.

All forms of learning compared in this study are established forms of teaching that the students have been aware of since the beginning of their studies. The content of the courses was precisely standardised to ensure the greatest possible equality between the groups; the materials were strictly based on a structured subject catalogue and contained the same text, illustrations and case samples to minimise the number of confounding variables.

The materials were strictly checked for their comparability, using a detailed catalogue of learning objectives. All lecturers and student instructors were also equipped with precise instructions for the implementation of the content. The means of standardisation and unavoidable differences are shown in Table 1.

**Table 1:** Means of standardisation between the study branches

	E-learning	Lecture	Seminars	Self-instructed learning
Exams	Multiple choice exams, pre-test and post-test	Identical	Identical	Identical
Curriculum and learning objectives	Catalog of 40 learning objectives	Identical	Identical	Identical
Instructor guidelines	Strict instructions and preparation materials	Identical	Identical	Identical
Subgrouping	Four groups of 12–15 students	One group of students	Four groups of 12–15 students	Four groups of 12–15 students
Images, texts and animations	Provided on-line, exact same content	Provided embedded into presentation	Provided embedded into presentation	Not provided
Total time	180 min	180 min	180 min	180 min
Patient case assignments	Completed online	Completed in discussion within lecture	Completed in small groups, then discussed	Not provided

### Pre-study tests

There are no reliable studies on the distribution of learning styles within the specific student population of reformed study programs. Therefore, prior to the study, the individual learning style was determined using the Canfield Learning Styles Inventory (CLSI) [13] through an online questionnaire to detect imbalances in the students’ allocation to the study branches. The CLSI is an established and reliable tool used to determine learning style and is widely used in educational research [14–17]. The 2004 version of the questionnaire contains 30 questions and 21 variables, to which five learning styles can be assigned for each. Further, epidemiological data was collected. Results of the questionnaires or the resulting learning style had no influence on the allocation to a study branch.

### Randomisation

The allocation to the various branches of the study was carried out by randomisation and without any regard for preferred learning style or previous results in other exams. All students were anonymously assigned in advance, using a number (‘token’) that was used for identification purposes throughout the study. Randomisation was achieved using the RAND function of Microsoft Excel 2010 (Microsoft, Redmond, USA). Instructors had no access to the token codes during or after the study.

### Context of the study

A regular course in the third year of the curriculum of endocrine pharmacology was chosen for the study. In the course, the students primarily studied treatment guidelines for *diabetes mellitus*, type II.

The course content included pathophysiology, epidemiology, pharmacokinetics, indications and contraindications of used pharmaceutics, drug interactions and therapeutic application. The second part involved the practical application of the diagnostic algorithm in the treatment of patients with *diabetes mellitus* in case studies and included the processing of case studies according to the guidelines of the German Diabetes Association [18]. Case studies and the materials used were also identical for all groups.

### Study branches

All students in the third study year of the Aachen model curriculum were randomly assigned to one of four study branches: two instructed (SE and VL) and two self-instructed (EL and SL) branches. The compared forms of learning were:

- a combined seminar in small groups with case discussion in single groups (‘SE’ group)
- a classical lecture in the auditorium (‘VL’ group)
- an e-learning course (self-instructed, self-taught online course) (’EL’ group)
- a non-guided, self-instructed reference group (‘SL’ group)

#### Lecture (VL)

The students participated in a traditional lecture in the auditorium. The presentation was guided by the course-instructor, an experienced lecturer in pharmacology. The professor had been teaching this topic in the course for several years. The lecture was given ex-cathedra, supplemented by a presentation with Microsoft PowerPoint. The presentation contained the same animations and illustrations as in the EL and SE groups. The practical case studies were discussed and explained by the professor.

#### Seminars (SE)

This study branch consisted of four groups of 13–15 students, guided by an experienced tutor throughout the seminar. All tutors were experienced scientists from the Institute of Pharmacology, who had been teaching in this field for years. The presentation used was the same as that of the lecture, as were the case studies released at the end for discussion in the plenary.

#### E-learning (EL)

The students participated in a linear structured online course, based on Moodle web-based learning software. The course included a series of 35 pages in three chapters, each ending with a self-assessment in the form of a quiz. The sites were supplemented by multimedia elements (five animations of pharmacokinetics as Shockwave Flash Files [see Additional files 1, 2, 3 and 4] and 12 pictures and diagrams). The course was carried out on individual computers within the university with Microsoft Windows XP and Mozilla Firefox 10.2. Participants took additional notes during the processing of the course with a prepared worksheet.

#### Non-guided self-instruction (SL)

Students in reformed study programs are generally encouraged to undergo a very individual, self-directed learning style from the outset. In order to assess this particular learning style, students in this group were only instructed minimally. The students were divided into groups of 13–15 and assigned to individual rooms. The students received a copy of the subject-catalogue with the formulated learning objectives (e.g. ‘The student should know the pharmacodynamics of Metformin’). This standardised subject-catalogue was also used in building all learning materials for the other groups.

The students were only told that they should follow the subject-catalogue. The students were free to choose which method they would use to learn the subject matter: they had a sufficient number of books, guidelines and internet access. They were also free to choose from all the materials provided. All groups were supervised by a tutor to ensure a serene learning environment.

A group of four general study supervisors was present at all times to answer any organisational questions.

### Data acquisition

A variety of established test procedures was used to compare the different study branches. The individual progress of students was measured before and after their respective learning modules. These tests were based on a 24-item multiple-choice questionnaire consisting of seven knowledge questions of low, medium and high difficulty respectively. Each question included five possible answers, of which only one was correct. An answer of ‘uncertain’ was offered to minimise the influence of random answers. Then, five questions were asked concerning the individual’s assessment of learning progress and preparedness for practical application. The participants had 60 s to answer each question. The full questionnaire can be accessed Additional files 5 and 6 online.

After four weeks, a voluntary online follow-up was offered to all participants to capture the general satisfaction and course experience of the students, as well as to detect methodical weaknesses and difficulties that might be overlooked by conventional test procedures. Using qualitative data analysis/tagging procedures, complementary aspects of free-text comments were covered. All content, materials and test-tools were evaluated in advance by independent professional representatives and were tested on small groups of students. The information and comments gathered contributed to the optimisation of materials.

### Statistical analysis software

Descriptive statistics were summarised as means, standard deviations, medians and percentages as appropriate. The determination of significant differences between pre- and post-tests was achieved using a student’s t-test.

An ANOVA (analysis of variance) was carried out to determine the influence of the assigned study branch to the test results. To detect inhomogeneity within the study population, a bivariate Chi-squared analysis was carried out; Cramer’s V was used for interpretation, as we faced a non-rectangular (1*k) table.

The statistical tests were performed with SAS, version 9.2 (SAS Institute Inc., Cary, USA), STATA, version 13 (StataCorp LP, College Station, USA) and Microsoft Excel 2010 (Microsoft, Redmond, USA). A qualitative data analysis was performed using the computerised tagging system WeftQDA, version 1.0.1 (Alexander Frank Fenton, Berlin).

The digitalisation of the questionnaires was carried out electronically through a scanner-based registration system. The online surveys were created using the LimeSurvey system version 0.58 (The LimeSurvey Project Team).

## Results

Of 244 initially registered participants, 223 participated on the test day. The main reason for not participating was acute sickness. One person retired during test participation because of an acute illness (see Fig. 1).

The study population included all 244 students in their third academic year. The average age was 23.2 years, and the percentage of women was 68.16 (Table 2). The students had been studying for an average of 3.1 years at the faculty, and 99.9 % took part in the course for the first time. Concerning personal preference for a certain learning style, the majority (38.78 %) of the participants chose E-learning.

**Table 2:** Main characteristics of the study population (*n* = 244)

Gender	Female	*n* = 167	68.16 %
	Male	*n* = 77	31.43 %
Age groups	19–22 years	*n* = 132	53.88 %
	23–24 years	*n* = 46	18.78 %
	25–26 years	*n* = 14	5.71 %
	27–28 years	*n* = 32	13.06 %
	Over 28 years	*n* = 14	5.71 %
	No answer	*n* = 7	2.86 %
Personal preference *(“What learning form would you choose, if you could?”)*	E-learning	*n* = 95	38.78 %
	Self-instructed learning	*n* = 44	17.96 %
	Seminar group	*n* = 56	22.86 %
	Lecture	*n* = 49	20.00 %
	No answer	*n* = 1	0.41 %

**Fig. 2:**
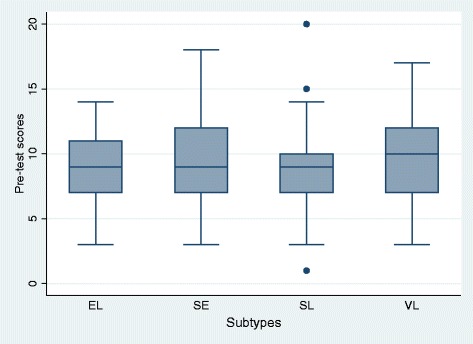
Achieved test scores in Pre-test

**Fig. 3:**
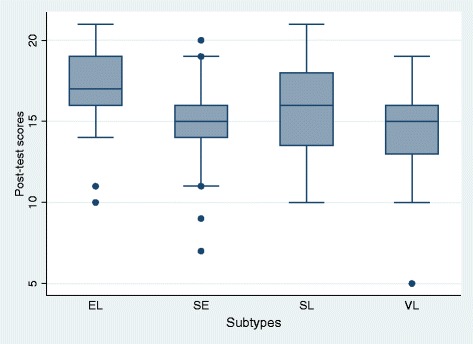
Achieved test scores in Post-test

In the pre-test, the groups were relatively homogeneous (Fig. 2), with results ranging from 8.81 obtained points in the group of self-instructed learning up to 9.24 points in the group that participated in a seminar (Table 3). After each course, the post-test was performed. All students showed notable improvements compared to the pre-test results (Fig. 3). Participants in the non-self-instructed learning group reached scores of 14.71 (seminar) and 15.81 (self-learners), but the group of self-instructed learners reached higher scores of 17.23 points (E-learning).

**Table 3:** Achieved test scores (each correct question scored 1 point)

Pre-test				Post-test		
	Mean	Standard deviation	Standard error of the mean	Mean	Standard deviation	Standard error of the mean
E-learning (EL)	9.08	2.69	0.344	17.23	2.21	2.206
Seminar (SE)	9.24	3.21	0.433	14.71	2.42	1.983
Self-instructed learning (SL)	8.81	3.23	0.448	15.81	2.81	2.192
Lecture (VL)	9.78	3.48	0.474	14.37	2.76	1.956

An ANOVA was carried out to determine the influence of the assigned study branch to the test results. The calculated model showed a high coefficient of determination of adj. R^2^ = 0.42 and high effect sizes, as shown in Table 4, both for the complete calculated model and for the partial models ‘subtype’ and ‘pre-test scores’. A Bartlett’s test for equal variances used to test for homogeneity of variance resulted in *p* = 0.234.

**Table 4:** Specifications of the calculated ANOVA eta squared

Effect size	
Source	Eta squared
Complete Model	0.43 (*p* < .001)
Subtype	0.26 (*p* < .001)
Pre-test scores	0.32 (*p* < .001)
Coefficient of determination	
Adjusted R^2^	0.4240
Homogeneity of variance	
Bartlett’s test for equal variances	*p* = 0.234

**Fig. 4:**
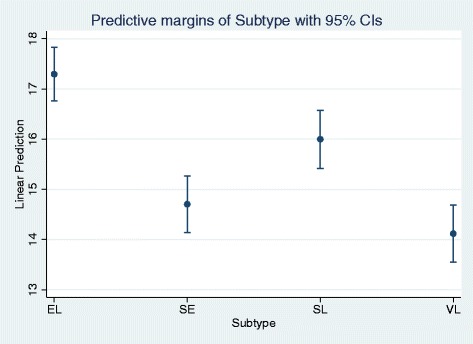
Prediction of post-values depending on subtypes (study branches)

The results of the calculated ANOVA are shown in Table 5. Comparison of the different study branches show significant differences between E-learning and Seminar (*p* < 0.001), E-learning and Self-Study (*p* < 0.05), E-learning and Lecture (*p* < 0.001) and Self-Study and Lecture groups (*p* < 0.05) (Table 5). Results of the calculated predictive margin are displayed in Fig. 4, which shows the influence of the study branch on the post-value. Here again, the self-instructed learning methods EL and SL showed higher predicted scores.

**Table 5:** Results of the ANOVA (predicted scores in the post-test)

	E-learning (EL)	Seminar group (SE)	Self-study (SL)
Seminar group (SE)	−2.52 *p* = 0.000		
Self-study (SL)	−1.42 *p* = 0.021	1.10 *p* = 0.162	
Lecture (VL)	−2.86 *p* = 0.000	−0.34 *p* = 1.00	−1.44 *p* = 0.025

The paired t-test showed that the mean post values of all pre-values differed from each other significantly at a level of (*p* < .001) (see Table 6).

**Table 6:** Paired Student’s t-test (*p* < .001)

Values	Mean scores	Standard deviation
Pre-test	9.226	3.150
Post-test	15.577	0.187

*p* < .001; *t* = −31.204; 95 % confidence interval

The multiple-choice items of the questionnaire also included the personal self-assessment of knowledge. This was determined using a Likert-scale from 1 (very good knowledge) to 5 (no knowledge). Here too, we found homogeneous self-assessments in the pre-test with mean values of 3.76 (seminar) and 3.92 (self-learner). All groups improved significantly in the post-test regarding their self-assessment, led by the e-learning group, whose self-assessment improved by 2.36 (see Table 7).

**Table 7:** Personal self-assessment (“How would you describe your level of knowledge about drug therapy for diabetes mellitus at the present time?” Scored 1 (very high level of knowledge) to 5 (I have no knowledge)

	Pre-test		Post-test	
	Mean	Standard deviation	Mean	Standard deviation
E-learning (EL)	3.91	0.58	2.36	0.58
Seminar (SE)	3.76	0.50	2.47	0.60
Self-instructed learning (SL)	3.92	0.68	2.77	0.67
Lecture (VL)	3.80	0.73	2.69	0.58

To determine inhomogeneity within the population in advance, the learning style of the population was additionally registered through an online questionnaire. The test was performed using the CLSI [13]. The questionnaires result in a test-specific X/Y score, which refers to the student’s preferred learning style (see Table 8).

**Table 8:** Distribution of learning styles within the study population

	X < -15	X -15 to 15	X > 15	Total
Y > 10	*n* = 7 Social/Applied	*n* = 41 Social	*n* = 39 Social/Conceptual	87
Y -10 to 10	*n* = 9 Applied	*n* = 45 Neutral	*n* = 29 Conceptual	83
Y < 10	*n* = 11 Independent/Applied	*n* = 19 Independent	*n* = 14 Independent/Conceptual	44
Total	27	105	82	214

We chose the bivariate Chi-square analysis method to detect accumulations of specific types of learning within the respective subtypes and the overall population The resulting Cramer V score (0.1827) was very weak and did show a significant level (*p* > .05) to prove that the assignment of subtypes did not depend on their respective learning style (see Table 9).

**Table 9:** Bivariate Chi-square analysis of the learning styles

	Learning style (Canfield Learning Styles Inventory)									
Subtype	SA	A	IA	S	N	I	SC	C	IC	Total
EL	2	2	3	9	16	5	6	9	6	58
	3.45	3.45	5.17	15.52	27.59	8.62	10.34	15.52	10.34	100
	28.57	22.22	27.27	21.95	35.56	26.32	15.38	31.03	42.86	27.1
SE	1	1	2	7	10	3	12	10	3	49
	2.04	2.04	4.08	14.29	20.41	6.12	24.49	20.41	6.12	100
	14.29	11.11	18.18	17.07	22.22	15.79	30.77	34.48	21.43	22.9
SL	1	1	4	10	10	6	12	6	3	53
	1.89	1.89	7.55	18.87	18.87	11.32	22.64	11.32	5.66	100
	14.29	11.11	36.36	24.39	22.22	31.58	30.77	20.69	21.43	24.77
VL	3	5	2	15	9	5	9	4	2	54
	5.56	9.26	3.7	27.78	16.67	9.26	16.67	7.41	3.7	100
	42.86	55.56	18.18	36.59	20	26.32	23.08	13.79	14.29	25.23
Total	7	9	11	41	45	19	39	29	14	214
	3.27	4.21	5.14	19.16	21.03	8.88	18.22	13.55	6.54	100
	100	100	100	100	100	100	100	100	100	100

Chi-squared = 21.4284; Cramer V = 0.1827; *p* = .613

### Follow-up

As a follow-up to the study, we conducted a standardised survey with 61 students of all subgroups to detect strengths and weaknesses of the study systematically. Participation in this survey was voluntary. These surveys were subjected to a qualitative data analysis. The largest criticisms were spatial problems or noise within the rooms (*n* = 14) and, due to organisational limitations, the late execution time of the study (*n* = 24). Nevertheless, many students endorsed the study and indicated a great personal learning effect (*n* = 21). Some exemplary statements from students are shown in Table 10.

**Table 10:** Exemplary statements from the follow-up survey (subgroup in brackets). Statements were translated from German into English

Positive statements	Negative statements
*Instructor quality:* “I was very happy with my seminar, the instructor was one of the best I’ve had so far, so I learned a lot.” “I really liked the lecture by the professor.” “The e-learning module was excellent and I wished more subjects would be taught in this way.” “Using the catalogue of learning objectives helps me to focus on the important aspects of the matter.” *Personal reflection:* “It was useful to find out what learning style you have, I would have preferred to know it before the study.” “I think the project is useful and important.” *Organisation:* “The study was well organised.” “I really liked the uncomplicated planning and execution of the experiment.”	*Environment and timing:* “The study was conducted too late in the day, I could not concentrate any more.” “You cannot learn all the names of the antidiabetics within this short amount of time.” “The room was too small and the air quality was low.” “It was too noisy to focus on your materials.” *Priming:* “Because of the questions of the pre-test, I focused more on these aspects so this could distort the results.” “I could answer a lot of the questions beforehand, so I did not notice a lot of improvement.” *Organisation:* “I did not like the random assignment to a subgroup, because the topic is very important and the assigned group is not my personal preference.”

## Discussion

Our study showed some surprising results. It could be shown clearly that the participants achieved significantly better results through modern self-instructed methods than through conventional methods.

In particular, the good performance of the EL group is remarkable. Recent findings by Augustin [15] reveal additional aspects that could have contributed to the success of the e-learning group: the group used the concept of ‘active recall’ – meaning participants were instructed to take notes during the study. Furthermore, during implemented quizzes, participants were given direct feedback as to whether their answers were correct or not. This was also emphasised by the results of the conducted ANOVA, that showed significant differences between EL and SE (*p* < 0.001) and EL and SL (*p* < 0.05).

As Augustin concluded, a “[…] significant contributor to the testing effect is initial feedback to teach the student whether an answer was correct or incorrect” [19]. The two self-instructed methods (in particular, the EL group) required more time for preparation, but could be utilised for several years. In addition, the students’ high level of satisfaction with this learning method is emphasised by our study.

The e-learning group assessed itself as being the best at the end of the study with an average of 2.37 points on the Likert scale; further, the results of this group’s post-tests, with an average of 17.23 points, were the highest. In addition, 38.78 % of the students showed a preference for e-learning modules in advance of the study, which shows a generally high acceptance of the learning form.

This method will be used increasingly in the near future within our faculty and the reformed study programme.

Non-self-instructed learning methods have a crucial disadvantage in the high, permanent cost of qualified staff and premises. However, they provide the significant benefit that the students can directly respond to and request feedback.

Surprisingly, the group that was the least instructed, and was initially planned as a reference group (SL), exceeded both teacher-instructed groups. From our point of view, its strong performance as the second strongest group could be explained in two ways: 1) as the very complex subjects of the courses cannot be fully taught in classrooms, students of medical courses are accustomed to concentrated individual work; and 2) subjects such as those in clinical pharmacology, who face a high amount of knowledge that has to be memorised, are predestined for self-instructed work. The initial analysis of personal learning styles using the CLSI showed a notable accumulation of learning styles within the Social/Conceptual area. The most common type of learner, “Social” – “prefers extensive opportunities to interact with peers and instructors” and “instruction involving small groups and teamwork will create the closest match” [10]. Another interesting finding of the study is that although nearly 40 % of the students show a social learning style (at least partially), the highest scores were achieved in the study branches where less social interaction is required. Although no direct link between learning style and results can be established, this might show that although mostly social learning forms are preferred, self-instructed learning forms remain the most effective way to learn complex theoretical subjects, such as clinical pharmacology.

However, our study also showed some limitations. Although the greatest effort was spent to standardise the learning conditions and materials, there remain confounders that could not be eliminated. With learning methods that include a social interaction, despite limitations of the material and the precise instruction of the teachers, one cannot entirely exclude the mention of other topics and issues. Moreover, the individual quality of the lecturer or teacher affects the results of the study. We have met these requirements with explicit instructions and limitations for the teachers.

Also, although a high number of knowledge questions within Pre-test and Post-test is desirable, we had to limit the sample size due to time restrictions. This may limit the power of the findings of this study. Due to the legal restrictions of the Faculty of Medicine, we were not allowed to link study dropouts to their participation within the pre-study tests, like Canfields Learning Style Inventory, etc. Therefore, no intention-to-treat analysis could be carried out. Further, the interpretation of the results remains limited to the assessed contents, namely the field of teaching clinical pharmacology. Whether these results can be directly transferred to other aspects of teaching where more direct instruction is needed (e.g. clinical examination courses, anatomy), will be the subject of further studies.

## Conclusion

This study demonstrated the importance of regular and continuous research when producing and implementing reformed medical curricula. We showed that self-directed learning outperformed direct instruction in the environment of a modern, hybrid medical curriculum. Although all examined methods of instruction reached high levels in post-test scores and student acceptance, the self-instructed study branches particularly showed promising cost-to-benefit ratios. The question of whether study-phases carried out individually strictly based on catalogues of learning objectives with subsequent success control should be integrated more frequently in medical curricula should be addressed by further research.

These results will influence the further development of the reformed study program in Aachen. Currently, several similar e-learning-based projects are in preparation or are already being performed. The development and creation of new and innovative learning products are promoted across institutions by the faculty.

## Abbreviations

ANOVA, analysis of variance; CLSI, canfield learning style inventory; EL, e-learning (abbreviation used within the study); GDA, German Diabetes Association; MEQ, modified essay question; OSCE, objective structured clinical examination; OSPE, objective structured practical examination; PBL, problem based learning; RWTH, Rheinisch-Westfälische Technische Hochschule; SE, seminar group (abbreviation used within the study); SL, self-learning (abbreviation used within the study); VL, vorlesung (lecture, abbreviation used within the study); WBL, web based learning.

## Additional files

Additional file 1: Type II diabetes mellitus development. Animation: The development of diabetes mellitus Type II within the context of lifestyle choices (study material). (SWF 141 kb) Additional file 2: Sulfonylurea stimulation of insulin secretion. Animation: The pharmacological agent Sulfonurea modulating insulin secretion (study material). (SWF 1148 kb) Additional file 3: Cellular mechanisms of Metformine. Animation: The pharmacological agent Metformine and its cellular mechanisms (study material). (SWF 2343 kb) Additional file 4: GLP-1 agonists and DPP-4 inhibitors. Animation: GLP-1 agonists and DPP-4 inhibitors and their role on insulin secretion (study material). (SWF 503 kb) Additional file 5: Full questionnaire of Pretest and Posttest. Full text of the used questionnaire in German language. (PDF 53 kb) Additional file 6: Full questionnaire of Pretest and Posttest (Translation). Full text of the used questionnaire in English language (not used within the context of the study, translated for this publication). (PDF 974 kb)
