# Erratum to: Self-directed learning can outperform direct instruction in the course of a modern German medical curriculum - results of a mixed methods trial

**DOI:** 10.1186/s12909-016-0790-2

**Published:** 2016-10-11

**Authors:** Arne Peine, Klaus Kabino, Cord Spreckelsen

**Affiliations:** Department of Medical Informatics, RWTH Aachen University, Faculty of Medicine, Pauwelsstr. 30, 52074 Aachen, Germany

## Erratum

After publication of the original article [1], the authors wish to correct the ‘Author’s contributions’ section in order to clarify the unspecific wording used to declare the contribution of Cord Spreckelsen. The original section, containing “AP wrote the manuscript with contributions from CS”, should have been published as follows:

“AP and CS developed the study concept. AP performed the data collection and follow-up analysis. AP wrote the manuscript. The manuscript was critically revised by CS. AP, CS and KK read and approved the final manuscript.”
