# Supplementary material for: Self-directed learning can outperform direct instruction in the course of a modern German medical curriculum - results of a mixed methods trial
Source: BMC Med Educ. 2016 Jun 3;16:158. doi: 10.1186/s12909-016-0679-0 (PMC4891889; doi:10.1186/s12909-016-0679-0)
Supplement: Additional file 5: — Full questionnaire of Pretest and Posttest. Full text of the used questionnaire in German language. (PDF 53 kb) [file 12909_2016_679_MOESM5_ESM.pdf]

Bitte tragen Sie Ihre Antworten auf dem beiliegenden Blatt ein.

Bitte beachten Sie: Eine richtige Antwort bringt einen Punkt, eine falsche Antwort einen Punkt Abzug! Sie haben bei allen Fragen auch die Möglichkeit „weiß nicht“ anzukreuzen. Diese wird neutral gewertet und führt nicht zu Punktabzug. Es kann daher in manchen Fällen sinnvoll sein, diese Option zu wählen.

Die ersten drei Fragen sind statistischer Natur und werden nicht bewertet.

1.
Wie würden Sie ihren Kenntnisstand zum jetzigen Zeitpunkt bezüglich Diabetes mellitus allgemein beschreiben?

A) Sehr gut
B) Eher gut
C) Durchschnittlich
D) Eher geringe Kenntnisse
E) Ich habe keine Vorkenntnisse.

2.
Wie würden Sie ihren Kenntnisstand zum jetzigen Zeitpunkt bezüglich medikamentöser Therapie des Diabetes mellitus beschreiben?

A) Sehr gut
B) Eher gut
C) Durchschnittlich
D) Eher geringe Kenntnisse
E) Ich habe keine Vorkenntnisse.

3.
Wie würden Sie ihren Kenntnisstand zum jetzigen Zeitpunkt bezüglich Diagnostik und Klassifikation des Diabetes mellitus beschreiben?

A) Sehr gut
B) Eher gut
C) Durchschnittlich
D) Eher geringe Kenntnisse
E) Ich habe keine Vorkenntnisse.

4.
Mit welcher der folgenden Folgeerkrankungen wird Diabetes mellitus, Typ 2 am wenigsten in Verbindung gebracht:

A) Nierenschäden
B) Herzinfarkte
C) Schlaganfälle
D) Allergien
E) weiß nicht

5.
Welche der folgend genannten Medikamente setzt man nicht zu einer Behandlung von Diabetes mellitus ein?

A) Sulfonylharnstoffe
B) Alpha-Glucosidase-Hemmstoffe
C) Insulin-Reuptakeinhibitoren
D) Biguanide
E) weiß nicht

6.
Welcher Begriff passt am besten in die Lücke? ***Die Zerstörung der Inselzellen beim Typ 1-Diabetes ist vor allem ____________ .***

A) immunologisch bedingt
B) chemisch bedingt
C) noradrenerg bedingt
D) psychologisch bedingt
E) weiß nicht

7.
Welche Aussage zum Diabetes mellitus, Typ 2 trifft zu?

A) Diabetes spielt in der Genese von Schlaganfällen keine Rolle.
B) Die weltweite Prävalenz des Diabetes mellitus steigt kontinuierlich an.
C) Der wichtigste Faktor in der Entstehung von Diabetes ist die Vergiftung mit Schwermetallen durch das Trinkwasser.
D) Diabeteserkrankungen treten nur im Erwachsenenalter auf.
E) weiß nicht

8.
Sulfonylharnstoffe bezeichnet man auch als insulinotrope Antidiabetika. Warum?

A) Sie werden zumeist in den Tropen eingesetzt.
B) Sie sind der chemischen Struktur von Insulin sehr ähnlich.
C) Sie beeinflussen die Inotropie von Muskelgewebe.
D) Sie fördern die Freisetzung von Insulin durch die Bauchspeicheldrüse.
E) weiß nicht

9.
Was bezeichnet man als Inkretin-Effekt?

A) Die intraoperative Erhöhung der Körpertemperatur nach Metformingabe
B) Die stärkere Insulinfreisetzung nach oraler Glucosegabe im Vergleich zur i.v. Applikation
C) Die Gewichtszunahme nach Gabe von Inkretinen
D) Die Verstärkung von Entzündungsprozessen nach Gabe von Insulin
E) weiß nicht

10.
Welche Aussage zu Biguaniden (z.B. Metformin) ist richtig?

A) Biguanide wirken direkt in der Membran der Peroxisomen
B) Substanzgruppe der Wahl beim Diabetes, Typ 1
C) Bei Nierenfunktionsstörungen eher zu empfehlen
D) Laktazidosen sind eine gefürchtete Nebenwirkung
E) weiß nicht

11.
Welche der unten genannten Medikamente führt am ehesten zu Gewichtszunahme?

A) Exenatid
B) Acarbose
C) Pioglitazon
D) Miglitol
E) weiß nicht

12.
Als %HbA_{1c} bezeichnet man...

A) Den prozentualen Anteil des glykierten Hämoglobins am Gesamthämoglobin.
B) Den laborchemischen Parameter der endogenen Insulinsekretion.
C) Den prozentualen Anteil von glykiertem Albumin im Blut.
D) Die Hämoglobinalkylase 1c bei Diabetikern.
E) weiß nicht

13.
Welche der unten genannten Begriffe spielt bei der Genese des Diabetes mellitus Typ II die geringste Rolle?

A) Periphere Insulinresistenz
B) Sekundärversagen nach Jahren
C) Kompensatorische Hypoinsulinämie
D) Hyperglykämie
E) weiß nicht

14.
Welches Medikament zählt man zu den Alpha-Glucosidasehemmstoffen?

A) Miglitol
B) Pioglitazon
C) Tolbutamid
D) Repaglinid
E) weiß nicht

15.
Mit welchem Kanal interagieren Sulfonylharnstoffe?

A) ATP-abhängiger Kaliumkanal
B) AMP-abhängiger Calciumkanal
C) insulinabhängiger Kaliumkanal
D) spannungsgesteuerter Natriumkanal
E) weiß nicht

16.
Auf welches Organsystem wirkt GLP-1 (Glucagon-like Peptide 1) nach heutigem Erkenntnisstand nicht?

A) Darm
B) Gehirn
C) Niere
D) Pankreas
E) weiß nicht

17.
Ein Patient stellt sich bei seinem Hausarzt im Rahmen eines jährlichen „Check up" vor. Bisher sind keine Vorerkrankungen bekannt. Der beim nüchternen Patienten erhobene Blutglucosespiegel liegt bei 191 mg/dl . Welche Aussage trifft zu?

A) Ein Diabetes mellitus liegt vor.
B) Eine gestörte Glucosetoleranz (aber kein Diabetes) liegt vor.
C) Der Patient ist gesund. Er soll sich in einem Jahr zur Routinekontrolle wieder vorstellen.
D) Eine Ketoazidose liegt vor.
E) weiß nicht

Wahr oder falsch?

18.
Pioglitazon wirkt am PPARγ-Rezeptor (Peroxisom-Proliferator-aktivierten Rezeptor-gamma)

A) Die Aussage ist richtig.
B) Die Aussage ist falsch.
C) weiß nicht

19.
Die genetische Prädisposition von Typ-2-Diabetes mellitus ist gering.

A) Die Aussage ist richtig.
B) Die Aussage ist falsch.
C) weiß nicht

20.

Ein diagnostisches Kriterium des metabolischen Syndroms (IDF-Klassifikation) ist die bauchbetonte Adipositas.

A) Die Aussage ist richtig.
B) Die Aussage ist falsch.
C) weiß nicht

21.

Metformin ist ein Alpha-Glucosidasehemmstoff.

A) Die Aussage ist richtig.
B) Die Aussage ist falsch.
C) weiß nicht

22.

Exenatid bezeichnet man auch als Insulin-Sensitizer.

A) Die Aussage ist richtig.
B) Die Aussage ist falsch.
C) weiß nicht

23.

Patrick Müller (24 Jahre) wird mit dem Krankenwagen nach einem „Kreislaufkollaps" in die Klinik eingeliefert. Er klagt schon seit 24 Stunden über Schwindel und Übelkeit. Sie messen bei dem Patienten einen Blutzuckerspiegel von 92 mg/dl. Welche Aussage trifft zu?

A) Der Blutzucker liegt im normwertigen Bereich. Die Suche nach der Kollapsursache sollte sich auf andere Bereiche konzentrieren.
B) Bei einem solchen Blutglucosespiegel sollte sofort Glucose i.v. infundiert werden.
C) Der Patient ist mit hoher Wahrscheinlichkeit Diabetiker.
D) Der Patient simuliert und sollte nicht weiter untersucht werden.
E) weiß nicht

24.

Eine 57-jährige Patientin kommt im Rahmen einer Routinekontrolle in Ihre Sprechstunde. Sie hat einen Bauchumfang von 110 cm an der weitesten Stelle. Weiter liegen bei ihr ein Hypertonus und ein diagnostizierter Diabetes mellitus Typ 2 vor, die Sie beide seit Jahren medikamentös behandeln. Welche Aussage trifft zu?

A) Folgeerkrankungen (z.B. diabetische Retinopathien) sind sehr selten.
B) Die Kriterien für die Diagnose eines metabolischen Syndroms gemäß Definition der International Diabetes Foundation sind erfüllt.
C) Die o.g. Kombination der Risikofaktoren führt nicht zu einer höheren Rate an Herzinfarkten.
D) Es sollte sofort eine ERCP (Endoskopisch retrograde Cholangiopankreatikographie) zur weiteren Abklärung folgen.
E) weiß nicht
