# Supplementary material for: Self-directed learning can outperform direct instruction in the course of a modern German medical curriculum - results of a mixed methods trial
Source: BMC Med Educ. 2016 Jun 3;16:158. doi: 10.1186/s12909-016-0679-0 (PMC4891889; doi:10.1186/s12909-016-0679-0)
Supplement: Additional file 6: — Full questionnaire of Pretest and Posttest (Translation). Full text of the used questionnaire in English language (not used within the context of the study, translated for this publication). (PDF 974 kb) [file 12909_2016_679_MOESM6_ESM.pdf]

This is a translated version of the original questionnaire.
Only the validated German version has been used within the study.
It may be used for research and educational purposes.

Please enter your responses on the attached sheet.

Please note: You'll get points for correct answers, a wrong answer will deduct points! For any questions you also have the possibility to tick "Don't know". This is considered neutral and does not lead to point deduction. It may therefore be useful to choose this option in some cases.

The first <u>three questions</u> are statistic questions and will not count to your total points.

1.
How would you define your state of knowledge at the <u>present time</u> regarding <u>diabetes mellitus in general?</u>

A) Very good
B) Rather good
C) Average
D) Rather small knowledge
E) I have no prior knowledge.

2.
How would you define your state of knowledge at the <u>present time</u> regarding <u>drug treatment of diabetes mellitus</u>?

A) Very good
B) Rather good
C) Average
D) Rather small knowledge
E) I have no prior knowledge.

3.
How would you define your state of knowledge at the <u>present time</u> regarding <u>diagnosis and classification of diabetes mellitus</u>?

A) Very good
B) Rather good
C) Average
D) Rather small knowledge
E) I have no prior knowledge.

4.
Which of the following illnesses is brought <u>least</u> into connection with diabetes mellitus type II?

A) Kidney damage
B) Heart attacks
C) Strokes

D) Allergies
E) Don’t know

5.
Which of the following drugs are not used in treating diabetes mellitus?

A) Sulphonylureas
B) Alpha-glucosidase inhibitors
C) Insulin Reuptake Inhibitors
D) Biguanides
E) Don’t know

6.
What term best fits into the gap? *The destruction of the islet cells in type I diabetes is_____.*

A) immunologically related
B) chemically related
C) a noradrenergic reaction
D) a psychological reaction
E) Don’t know

7.
Which statement about the diabetes mellitus type II is true?

A) Diabetes plays no role in the development of cerebral strokes.
B) The global prevalence of diabetes mellitus is rising steadily.
C) The most important factor in the development of diabetes is the poisoning with heavy metals from drinking water.
D) Diabetes cases only occur in adults.
E) Don’t know

8.
Sulphonylureas are also referred to as insulinotropic antidiabetic agents. Why?

A) They are mostly used in the tropics.
B) They are very similar to the chemical structure of insulin.
C) They influence the inotropy of muscle tissue.
D) They increase the release of insulin from the pancreas.
E) Don’t know

9.
What is referred to as “Incretin effect”?

A) The intra-operative increase of body temperature due to metformin administration
B) The increased insulin release after oral glucose administration in comparison to the i.v. application
C) The increase in body weight after administration of incretins
D) The reinforcement of inflammatory processes after administration of insulin
E) Don’t know

10.

Which statement is correct about biguanides (e.g. Metformin) ?

A) Biguanides act directly in the membrane of the peroxisomes
B) First medication of choice in patients with diabetes, type I
C) Advised in patients with renal impairment
D) Lactic acidosis is a dreaded side effect
E) Don't know

11.

Which of the below mentioned drugs will most likely lead to weight gain?

A) Exenatide
B) Acarbose
C) Pioglitazone
D) Miglitol
E) Don't know

12.

The term "%HbA1c" designates…

A) The fraction of glycated hemoglobin on total hemoglobin.
B) The chemical parameters of endogenous insulin secretion.
C) The percentage of glycated albumin in the blood.
D) The hemoglobinalkylase 1c in diabetic patients.
E) Don't know

13.

Which of the below factors plays the lowest role in the development of diabetes mellitus type II?

A) Peripheral insulin resistance
B) "Secondary failure" after years
C) Compensatory hypoinsulinemia
D) Hyperglycaemia
E) Don't know

14.

Which drug is an alpha glucosidase inhibitor?

A) Miglitol
B) Pioglitazone
C) Tolbutamide
D) Repaglinide
E) Don't know

15.

Sulfonylureas interact with the…

A) ATP-sensitive potassium channel
B) AMP-dependent calcium channel
C) Insulin-dependent potassium channel
D) Voltage-sensitive sodium channel
E) Don't know

16.

GLP-1 (glucagon-like peptides 1) do not interact with the following organ system (according to the current state of knowledge)

A) Gut
B) Brain
C) Kidney
D) Pancreas
E) Don't know

17.

A patient is seeing his family physician during a "check-up". There are currently no known medical conditions. The raised fasting blood glucose level is 191 mg/dl. Which of the following statements is true?

A) A diabetes mellitus can be diagnosed.
B) Impaired glucose tolerance (but no diabetes).
C) The patient is healthy. He should be checked again in a year.
D) A ketoacidosis.
E) Don't know

True or false?

18.

Pioglitazone acts on PPAR- γ -receptors (Peroxisome proliferator-activated gamma receptors).

A) The statement is correct.
B) The statement is incorrect.
C) Don't know

19.

The genetic predisposition of type 2 diabetes mellitus is low.

A) The statement is correct.
B) The statement is incorrect.
C) Don't know

20.

A <u>diagnostic criterion</u> of Metabolic Syndrome (IDF classification) is the abdominal obesity.

A) The statement is correct.
B) The statement is incorrect.
C) Don't know

21.

Metformin is an alpha-glucosidase inhibitor.

A) The statement is correct.
B) The statement is incorrect.
C) Don't know

22.

Exenatide is also referred to as an insulin sensitizer.

A) The statement is correct.
B) The statement is incorrect.
C) Don't know

23.

Patrick Müller (24 years), is brought to the hospital after a "circulatory collapse". He complains of dizziness and nausea during the last 24 hours. The raised blood glucose level is 92 mg/dl. Which of the following statements is true?

A) The blood glucose level is normal. You should go on with other diagnostic tests to find the reason for his complaints.
B) Such blood glucose levels should immediately be treated via an intravenous glucose administration.
C) The patient is likely a diabetic.
D) The patient is simulating his complaints and should not be diagnosed further.
E) Don't know

24.

A 57-year-old patient comes to a routine check to your practice. She has an abdominal circumference of 110 cm at the widest point. She also has been diagnosed with hypertonia and <u>diabetes mellitus type 2,</u> that has been treated with drugs for years. Which of the following statements is true?

A) Following disorders (e.g. diabetic retinopathy) are very rare.
B) The criteria for the diagnosis of metabolic syndrome are met in accordance with the definition of the International Diabetes Foundation.
C) The above combination of risk factors does not lead to a higher rate of heart attacks.
D) The patient should receive an ERCP (Endoscopic retrograde cholangiopancreatography) for further diagnosis.
E) Don't know
